# Sigma-1 Receptor: A Potential Therapeutic Target for Traumatic Brain Injury

**DOI:** 10.3389/fncel.2021.685201

**Published:** 2021-09-30

**Authors:** Mingming Shi, Fanglian Chen, Zhijuan Chen, Weidong Yang, Shuyuan Yue, Jianning Zhang, Xin Chen

**Affiliations:** ^1^Department of Neurosurgery, Tianjin Medical University General Hospital, Tianjin, China; ^2^Department of Neurosurgery, Tianjin Neurological Institute, Key Laboratory of Post-trauma Neuro-repair and Regeneration in Central Nervous System, Ministry of Education, Tianjin, China; ^3^Department of Neurosurgery, Tianjin Key Laboratory of Injuries, Variations and Regeneration of Nervous System, Tianjin, China

**Keywords:** sigma-1 receptor, calcium homeostasis, endoplasmic reticulum stress, excitotoxicity, apoptosis, inflammatory responses, traumatic brain injury

## Abstract

The sigma-1 receptor (Sig-1R) is a chaperone receptor that primarily resides at the mitochondria-associated endoplasmic reticulum (ER) membrane (MAM) and acts as a dynamic pluripotent modulator regulating cellular pathophysiological processes. Multiple pharmacological studies have confirmed the beneficial effects of Sig-1R activation on cellular calcium homeostasis, excitotoxicity modulation, reactive oxygen species (ROS) clearance, and the structural and functional stability of the ER, mitochondria, and MAM. The Sig-1R is expressed broadly in cells of the central nervous system (CNS) and has been reported to be involved in various neurological disorders. Traumatic brain injury (TBI)-induced secondary injury involves complex and interrelated pathophysiological processes such as cellular apoptosis, glutamate excitotoxicity, inflammatory responses, endoplasmic reticulum stress, oxidative stress, and mitochondrial dysfunction. Thus, given the pluripotent modulation of the Sig-1R in diverse neurological disorders, we hypothesized that the Sig-1R may affect a series of pathophysiology after TBI. This review summarizes the current knowledge of the Sig-1R, its mechanistic role in various pathophysiological processes of multiple CNS diseases, and its potential therapeutic role in TBI.

## Introduction

The Sigma-1 receptor (Sig-1R) is a transmembrane protein containing 223 amino acids and was cloned more than 20 years ago (Hanner et al., 1996). Structurally, the three-dimensional structure of the Sig-1R was elusive for many years, but the crystal structure of the human Sig-1R has been recently clarified (Schmidt et al., 2016). An examination of the molecular basis for receptor oligomerization and ligand recognition revealed a trimeric architecture with a signal-pass transmembrane topology in each protomer (Schmidt et al., 2016). The Sig-1R has been reported to be expressed in several tissues, such as those of the central nervous system (CNS), retina, liver, kidney, lung, and heart (Hayashi and Su, 2007; Bhuiyan and Fukunaga, 2011; Ellis et al., 2017). Particularly, the Sig-1R is broadly expressed in cells of the CNS and is involved in the pathogenesis of CNS diseases, including Alzheimer’s disease (AD; Lahmy et al., 2013), Huntington’s disease (HD; Ryskamp D. et al., 2017), Parkinson’s disease (PD; Francardo et al., 2014) and amyotrophic lateral sclerosis (ALS; Ono et al., 2014). At the cellular level, the Sig-1R is an endoplasmic reticulum (ER) chaperone protein that mainly resides at the mitochondria-associated ER membrane (MAM), a dynamic and multifunctional scaffold for enabling crosstalk between the ER and mitochondria (Hayashi and Su, 2007; Sun et al., 2017). Under physiological conditions, the Sig-1R binds with the chaperone-binding immunoglobulin protein (BiP)/glucose-regulated protein 78 (GRP78) to form a complex at the MAM. Intriguingly, upon the stimulation of agonists or stress, the Sig-1R dissociates from the BiP and subsequently interacts with type 3 inositol 1,4,5-trisphosphate receptor3 (IP3R3) or translocates from the MAM to other cellular compartments such as the plasma membrane, the ER membrane, and the nuclear envelope (Hayashi and Su, 2007). Sig-1Rs are then able to interact with various cellular interaction partners including ion channels, receptors, and kinases (Su et al., 2010). In addition, the Sig-1R can also translocate to the envelope of the nucleus where it binds to the inner nuclear envelope protein emerin and recruits several chromatin-remodeling factors to regulate gene transcription (Tsai et al., 2015). Hence, owing to its universal ligand recognition, specific cellular location, and multi-site translocation profile, the Sig-1R is able to act as a pluripotent modulator in diverse pathological conditions.

To date, Sig-1R involvement has been reported in multiple pathophysiological processes, including calcium homeostasis, ER stress, autophagy, excitotoxicity, mitochondrial dysfunction, and reactive oxygen species (ROS) scavenging. The Sig-1R is expressed in neurons, microglia, astrocytes, and oligodendrocytes in the nervous system. Numerous lines of preclinical evidence have demonstrated the Sig-1R exerts multiple neuroprotective roles in neuronal apoptosis, neuronal excitotoxicity, motoneuron degeneration, and neuroinflammation in various neurological disorders. Among the many neurological disorders, traumatic brain injury (TBI) is generally regarded as a major public health problem. Due to its rising prevalence, wide-ranging risk factors, and significant impacts on families and society, it has aroused the attention of researchers, clinical and social service workers, as well as government policymakers. Globally, more than 50 million individuals suffer from TBI each year, and it is predicted that approximately half of the world’s population will experience a TBI in their lifetime (Maas et al., 2017). TBI is recognized as a leading cause of death among young adults and a major cause of mortality and disability worldwide (Roozenbeek et al., 2013; Quaglio et al., 2017). The vast expenditures in clinical management of TBI patients and related socio-economic aspects have placed a heavy burden on medical systems and society; it is estimated that TBI costs $400 billion in global economy losses annually (Maas et al., 2017). TBI is particularly challenging because it is a highly heterogeneous injury and often results in complicated pathogenesis (Bramlett and Dietrich, 2015).

While promising therapeutic approaches in preclinical studies have been developed for TBI, no effective therapeutic approaches have been successfully implemented to improve the prognosis of patients (McConeghy et al., 2012; Diaz-Arrastia et al., 2014; Gruenbaum et al., 2016). TBI comprises both initial primary and delayed secondary brain injury, in which primary brain injury causes irreversible and untreatable brain damage due to the mechanical injury, whereas secondary brain injury refers to further damage due to the injurious biochemical cascades induced by primary injury (Lingsma et al., 2010). Secondary brain injury involves cellular calcium homeostasis imbalance, glutamate excitotoxicity, endoplasmic reticulum (ER) stress, oxidative stress and mitochondrial dysfunction, apoptosis, and neuroinflammation (Lingsma et al., 2010; Sande and West, 2010; Nizamutdinov and Shapiro, 2017). As secondary brain injury occurs just hours to days following TBI, the timely and effective intervention of secondary injury at an early stage may effectively improve the prognosis of TBI patients. Based on recent advances in our understanding of pluripotent modulation of the Sig-1R in multiple pathological alterations following various CNS diseases, we propose that the Sig-1R may exert a critical effect on pathophysiological processes of TBI. In this review, we briefly summarize the recent advances in understanding of the Sig-1R and its mechanism in diverse pathophysiological conditions. We then discuss these emerging findings in the context of possible interrelationships between Sig-1Rs and pathogenesis of TBI, highlighting the Sig-1R as a potential therapeutic target for TBI.

## Sigma-1 Receptor Is Involved in Modulation of Endoplasmic Reticulum Stress

**FIGURE 1:**
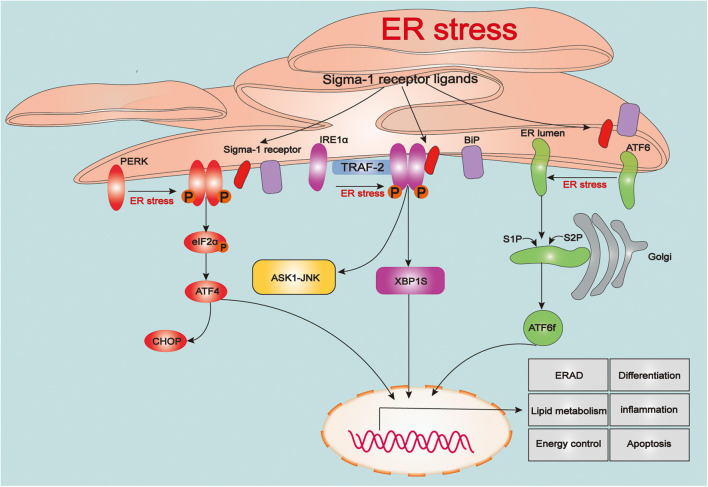
The role of sigma-1 receptor in regulating the unfolded protein response signaling. Upon the endoplasmic reticulum (ER) stress, three types of sensors of ER stress: protein kinase RNA-like ER kinase (PERK), inositol-requiring enzyme 1α (IRE1α) and activating transcription factor 6 (ATF6) are evoked and triggers a series of downstream signaling pathways that associated various pathophysiological processes including protein folding, lipid metabolism, energy control, differentiation, inflammation and apoptosis. Upon the ER stress or agonist stimulation, sigma-1 receptor (Sig-1R) dissociates from binding immunoglobulin protein (BiP) and immediately participates in regulating the unfolded protein response (UPR)-dependent signaling pathways.

The endoplasmic reticulum (ER) is the largest tubular-reticular organelle that spreads across the cell and plays a pluripotent and critical role in eukaryotic cells, including the storage and release of calcium, lipid synthesis, and intracellular signaling, as well as synthesis, folding, maturation, and trafficking of the cellular proteome (Rapoport, 2007; Phillips and Voeltz, 2016). Accumulation of misfolded and unfolded proteins in the ER contributes to ER stress, which subsequently activates the unfolded protein response (UPR), an adaptive process linked to maintenance of cellular proteostasis (Walter and Ron, 2011). The UPR is a sophisticated response linked to protein folding, and also participates in various physiological processes, such as lipid metabolism, energy control, differentiation, and inflammation (Rutkowski and Hegde, 2010; Wang and Kaufman, 2012). However, intense or prolonged ER stress in cells caused by severe damage such as TBI results in excessive UPR and calcium overload in mitochondria, triggering a series of cellular death processes (Tabas and Ron, 2011; Wang and Kaufman, 2016). With the ER stress or ligand stimulation, the Sig-1R dissociates from the BiP to modulate three types of sensors of ER stress, protein kinase RNA-like ER kinase (PERK), inositol-requiring enzyme 1α (IRE1α), and activating transcription factor 6 (ATF6), to regulate UPR signaling pathways (Figure 1).

### Sigma-1 Receptor and Protein Kinase RNA-Like Endoplasmic Reticulum Kinase-Mediated Endoplasmic Reticulum Stress

Protein kinase RNA-like ER kinase (PERK) is suppressed by BiP under physiological conditions, whereas it activates from restriction of BiP and initiates actions of dimerization and autophosphorylation upon ER stress. Activation of PERK phosphorylates downstream eukaryotic translation initiator 2α (eIF2α), which reduces protein synthesis and load on ER to inhibit stress. However, phosphorylation of eIF2α (p-eIF2α) also increases selective translation of mRNA activating transcription factor 4 (ATF4), which enters into nuclear and subsequently modulate genes expression involved in processes of the antioxidant response, amino acid metabolism and autophagy as well as apoptosis such as C/EBP-homologous protein (CHOP) (Hetz et al., 2015). Recently, several findings have reported that Sig-1R is involved in PERK/eIF2α/ATF4 pathway. Previous study demonstrated that overexpression of Sig-1R inhibited protein expression of p-PERK (Hayashi and Su, 2007). Under oxidative stress, RGC-5 cells were treated with (+)-pentazocine, a Sig-1R ligand, could significantly attenuate the mRNA expression level of PERK, ATF4 and CHOP as well as cells apoptotic death (Ha et al., 2011). *In vivo* stroke model, with using immunohistochemistry assays, it was shown that after treatment of a novel Sig-1R agonist, aniline derivative compound (Comp-AD) significantly reduced the expression of PERK (Morihara et al., 2018). In addition, recent study reported that Sig-1R agonist (PRE-084) decreased the proteins expression of p-PERK, ATF4 and CHOP at 3 days in bilateral common carotid artery occlusion (BCCAO) mouse model, whereas these effects were suppressed by Sig-1R antagonist (BD-1047) (Zhao et al., 2019). Meanwhile, proteins expression of p-PERK, p-eIF2α ATF4 and CHOP, TUNEL-positive cells and nuclear structural impairment in cortical neurons were significantly increased at 3 days after reperfusion in Sig-1R knockout (Sig-1R-KO)-BCCAO mice (Zhao et al., 2019). Sig-1R was also transcriptionally increased through PERK/eIF2α/ATF4 pathway due to there is an interaction between ATF4 and 5′ flanking region of Sig-1R gene in ER stress (Mitsuda et al., 2011). furthermore, it was found that a selective serotonin inhibitor (SSRI), Fluvoxamine, upregulated the expression of Sig-1R by directly increasing translation of ATF4 without activation of PERK/eIF2α/ATF4 signaling pathway (Omi et al., 2014). Thus, these findings above suggest that there is a close interrelationship between Sig-1R and PERK-mediated ER stress.

### Sigma-1 Receptor and Inositol-Requiring Enzyme 1α-Mediated Endoplasmic Reticulum Stress

The process of activating inositol-requiring enzyme 1α (IRE1α) is similar to PERK, in which it also undergoes its dimerization and trans-autophosphorylation. Activated IRE1α (p-IRE1α), owing to its endoribonuclease activity, which splices the mRNA encoding the transcription factor X-box binding protein 1 (XBP1) (Calfon et al., 2002). Spliced XBP1 (XBP1s) enters into nuclear and subsequently targets and favors the transcription of genes related to promotion of protein folding, ER-assisted protein degradation (ERAD), protein translocation and autophagy. Paradoxically however, interaction between p-IRE1α and adaptor proteins such as protein tumor necrosis factor (TNF) receptor-associated factor 2(TRAF2) triggers activation of JUN-terminal kinase (JNK) and apoptosis signal-regulating kinase 1 (ASK1) pathway related to apoptosis (Lee et al., 2003; Wang and Kaufman, 2016). IRE1α/XBP1s signaling is the most conserved UPR pathway in mammals, owing to its multi-functions on restoring ER proteostasis (Wang et al., 2014). In previous immunoprecipitation and ER stress studies *in vitro*, it was demonstrated that Sig-1R directly stabilized and folded structure of IRE1α (Mori et al., 2013). It was found that overexpressed Sig-1R in cardiomyocytes significantly increased the expression of p-IRE1α and XBP1s and promoted XBP1s nuclear trafficking under adaptive ER stress, whereas knockdown of Sig-1R inhibited these processes (Alam et al., 2017). However, excessive activation of p-IRE1α triggers a series of pro-apoptotic processes during prolonged and severe ER stress probably related to increasing expression of JNK and ASK1. It has been summarized that using Sig-1R ligands efficiently reduced the mRNA and protein expression of p-IRE1α suffering from prolonged and severe ER stress following cerebral ischemia injury; meanwhile, expression of p-IRE1α were significantly increased in (Sig-1R-KO)-BCCAO mice (Morihara et al., 2018; Zhao et al., 2019). Moreover, It has been reported that IRE1α/XBP1 signaling pathway activated by Toll-like receptor 4(TLR4)led to inflammation and innate immune responses (Martinon et al., 2010; Cho et al., 2013). It was shown that Sig-1R reduced production of inflammatory cytokine and mortality of mouse by inhibiting the endonuclease activity of IRE1α without affecting on classical inflammatory signaling pathway in a preclinical model of sepsis (Rosen et al., 2019). A recent study reported that fluvoxamine stimulation of Sig-1R attenuated ER stress primarily through IRE1α signaling in activated cardiac fibroblasts (Qu et al., 2021). Taking these findings together, we conjecture that Sig-1R constitutively regulates both mechanisms of adaptive response of the UPR (buffering the overload of misfolded proteins and evoking protective downstream signaling pathways) and excessive response of the UPR (triggering a series of pro-apoptotic and pro-inflammatory programs) under diverse pathophysiological conditions, all of which provide protective effects against cell damage.

### Sigma-1 Receptor and Activating Transcription Factor 6-Mediated Endoplasmic Reticulum Stress

Activating transcription factor 6 (ATF6) is a principal transducer of adaptive UPR response for sensing accumulation of misfolded proteins in ER (Adachi et al., 2008). Upon ER stress, ATF6 is transferred to Golgi, where it is cleaved by Site-1 protease(S1P)and Site-2 protease (S2P), releasing its cytosolic fragment (ATF6f). ATF6f is then transported into nuclear and considered as a transcription factor, in which it facilitates expression of genes related to counteraction of misfolded proteins in ER such as ER-associated protein degradation (ERAD), BiP and XBP1 (Bommiasamy et al., 2009). Studies have shown, in which Sig-1R agonist reduced mRNA expression of ATF6 in RGC-5 cells exposed to oxidative stress (Ha et al., 2011), and Sig-1R antagonist (NE-100) increased protein expression of cleaved ATF6 (p50) under tunicamycin-induced ER stress in HT22 cells (Ono et al., 2013). Furthermore, it was found that Sig-1R overexpression suppressed ER stress-induced ATF6 expression in CHO cells (Hayashi and Su, 2007). Knockout of Sig-1R contributed to an increase of expression of ATF6 after 1 year in neural retina of mice (Ha et al., 2014). However, it was reported in recent study that there was no change in ATF6 expression in Comp-AD treated mice under ischemic conditions (Morihara et al., 2018). During ER stress, ATF6 plays an important role in facilitating protein folding, but why activating Sig-1R cannot promote its expression and activity. Further explorations are required in this regard.

### Sigma-1 Receptor and Endoplasmic Reticulum-Assisted Protein Degradation

It has been demonstrated that many signaling pathways are involved in modulating the accumulation of misfolded proteins in ER. In particular, ERAD through discarding and retro-translating such misfolded proteins into cytosol for ubiquitination, deglycosylation and proteolysis, operates as a primary pathway toward ER proteostasis (Lilley and Ploegh, 2004; Hwang and Qi, 2018). Previous studies have reported that Sig-1R regulates three UPR sensors and their downstream pathways to influence the expression of ERAD. It was shown that Sig-1R directly bound with Insig1 to form a complex which regulates functions of ERAD and ERAD-mediated degradation of the CGalT (Hayashi et al., 2012). The latest study manifested that Sig-1R in brown adipocytes acted as a substrate of ERAD and a vital regulator of Sel1l-Hrd1 ERAD-mediated ER-mitochondria crosstalk, and mitochondrial dynamics (Zhou Z. et al., 2020).

## Sigma-1 Receptor Exerts Pluripotent Regulatory Effects on Mitochondrial Function

**FIGURE 2:**
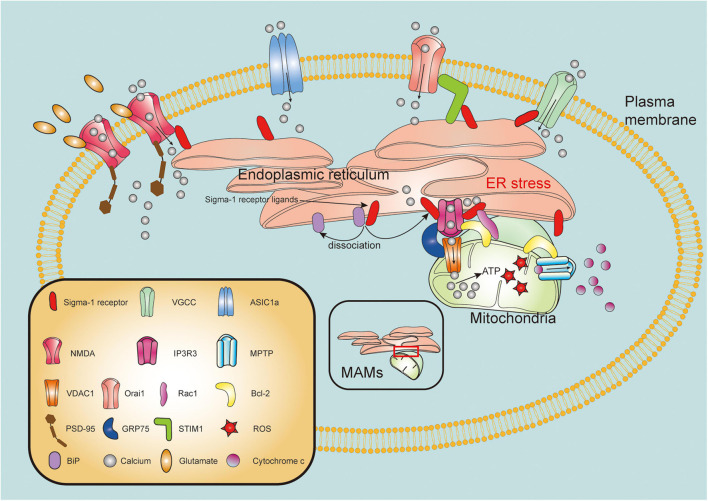
Molecular mechanisms of sigma-1 receptor in modulation of mitochondrial function and intracellular calcium homeostasis. Upon the ER stress or agonist stimulation, sigma-1 receptor (Sig-1R) dissociates from binding immunoglobulin protein (BiP) and binds to type 3 inositol 1,4,5-trisphosphate receptor (IP3R3), mainly resides at the mitochondria-associated membranes (MAMs). IP3R3s forms a tripartite complex with voltage-dependent anion-selective channel (VDAC) and glucose-regulated protein 75 (GRP75) to connect the ER and the mitochondria. Sig-1R activation facilitates calcium transfer from endoplasmic reticulum (ER) to mitochondria through IP3R3-GRP75-VDAC1 channel. Excessive ROS production promotes mitochondrial permeability transition pore (MPTP) opening, leading to release of cytochrome c from mitochondria to cytosol. Sig-1R may directly form a complex with IP3R, Bcl-2 and Ras-related C3 botulinum toxin substrate 1 (Rac1). Furthermore, Sig-1R can translocate from MAMs to plasma membrane where it directly or indirectly modulates intracellular calcium homeostasis by regulating activities of various plasma membrane elements including N-methyl-D-aspartate receptors (NMDARs), voltage-gated calcium channels (VGCCs), acid-sensing ion channel a (ASIC1a) and stromal interaction molecules 1 (STIM1)/Orai1 complex.

The mitochondria is one of the fundamental organelles in cells, related to critical functions in bioenergetics, metabolism, and cell survival/death (Harbauer et al., 2014). Mitochondrial dysfunction, as one of pathological hallmarks of TBI, is closely associated with neuronal necrosis, apoptosis, neuroinflammation, and autophagy following brain injury (Cheng et al., 2012). Strategies to maintain mitochondrial integrity and functions are therefore pivotal therapeutic targets for TBI. IP3R3s form a tripartite complex with outer mitochondria membrane (OMM)-located protein mitochondrial voltage-dependent anion-selective channel (VDAC) and glucose-regulated protein 75 (GRP75) to connect the ER and the mitochondria, thereby regulating calcium transfer from the ER into the mitochondria (Szabadkai et al., 2006; Cardenas et al., 2010). With ER stress or ligand stimulation, the Sig-1R dissociates from the BiP and subsequently interact with IP3R3s to guarantee activity and stability of this complex at the MAM (Hayashi and Su, 2007). Recently, it has been suggested that the Sig-1R not only clamps both the ER and mitochondria, but also has pluripotent regulatory effects on mitochondrial integrity and function (Figure 2).

Mitochondrial membrane potential (ΔΨm) is known to be a key factor to reflect the mitochondrial integrity, and dissipation of ΔΨm concomitantly destroys the mitochondrial integrity and induces cell apoptosis (Kadenbach et al., 2011). In mouse-cultured hippocampal HT22 cells, the Sig-1R agonist imipramine was observed to efficiently decrease tunicamycin-induced impairment of ΔΨm and cell death, whereas its effects were blocked by NE-100, a selective Sig-1R antagonist (Ono et al., 2012). Additionally, under oxygen- and glucose-deprived (OGD) conditions, agonism or overexpression of the Sig-1R in retinal ganglion cells (RGCs) significantly reduced the loss of ΔΨm and the increase of cytochrome c oxidase activity and caspase-3/7 activity, while these effects were also blocked by Sig-1R antagonists (Ellis et al., 2017).

It has been demonstrated that Bcl-2 protein family, which form complexes of mitochondrial permeability transition pore (MPTP) to regulate ΔΨm and maintain mitochondrial integrity, play a pivotal role in determining mitochondrial multiple death signaling pathways (Kwong and Molkentin, 2015; Bock and Tait, 2020). Under pathological conditions, both pro-apoptotic proteins Bax and Bak activate from restriction of pro-survival Bcl-2-like proteins, causing disruption of mitochondrial outer membrane and then leading to the release of cytochrome c and activation of caspase cascades (Youle and Strasser, 2008; McArthur et al., 2018). In oligomeric amyloid-β_25__–__35_ peptide (Aβ_25__–__35_)-injected mouse model of AD, it was observed that administration of Sig-1R agonists ANAVEX2-73 and PRE-084 not only inhibited Aβ_25__–__35_-induced elevation of lipid peroxidation levels and Bax/Bcl-2 ratio, as well as release of cytochrome c, all of which are related to mitochondrial integrity, but also corrected Aβ_25__–__35_-induced mitochondrial respiratory dysfunction and aberrant increase of reactive oxygen species (ROS) and apoptosis (Lahmy et al., 2014). Additionally, pretreatment with amyloid Aβ peptide in Alzheimer’s disease model, Sig-1R agonist OZP002 significantly lessened expression level of Bax in mouse hippocampus (Maurice et al., 2019). Conversely, significant decrease of both Bcl-2 mRNA and protein expression levels were found in retinas of Sig-1R KO mice (Ha et al., 2014). It was found that knockdown of Sig-1R promoted cellular apoptosis through down-regulating transcriptional expression of Bcl-2 *via* ROS/NF-κB pathway (Meunier and Hayashi, 2010). Recent study has also discovered that inhibition of Sig-1R could significantly upregulate expression levels of pro-apoptotic proteins such as Bax, Caspase-3 and Caspase-9 in BV-2 microglial cell induced by Methamphetamine (Shen et al., 2016). Ras-related C3 botulinum toxin substrate 1 (Rac1) as a member of the Rho family small GTPases, is highly expressed in CNS (Stankiewicz and Linseman, 2014). Rac1 involved in activation of phagocyte-like NADPH oxidase (Nox2) (Hordijk, 2006), plays critical role in neuronal migration, growth and morphogenesis (Watabe-Uchida et al., 2006; Xu et al., 2019). Intriguingly, it has been reported that interaction of mitochondrial Rac1 with Bcl-2 is implicated in generation of ROS, in which inhibition of formation of Rac1/Bcl-2 ameliorates neuronal oxidative stress damage following cerebral ischemia reperfusion (Pan et al., 2018). Previous study discovered that Sig-1R was involved in regulating hippocampal dendritic spine formation *via* TIAM1-Rac1GTP signaling pathway (Tsai et al., 2009). In further study with using immunoprecipitation techniques, it was shown that Sig-1R not only directly interacted with mitochondrial Rac1, but also assembled a complex with IP3R and Bcl-2 in the brain mitochondria (Natsvlishvili et al., 2015). Meanwhile, it was suggested in this study that Sig-1R-mediated Rac1 signaling promoted production of mild oxidative stress which is implicated in the regulation of neuroplasticity, apoptosis and autophagy (Natsvlishvili et al., 2015). Collectively, these findings above suggest that Sig-1R may directly form a complex with IP3R, Bcl-2 and Rac1, and Sig-1R ligands constitutively promote assembly and activation of this complex, maintaining mitochondrial integrity and regulating mitochondrial functions, as well as promoting cell survival (Figure 2). Furthermore, it was found that Sig-1R agonist SA4503 effectively rescued the reduced mitochondria size and promoted the expression levels of ER-mitochondria linkage proteins such as GRP75 and Mfn2 in cultured cardiomyocytes exposed to angiotensin II (Tagashira et al., 2013).

Dysfunction of mitochondria-induced overproduction of ROS is the primary pathological hallmark of postinjury following TBI (Abdul-Muneer et al., 2015). Increased accumulation of ROS in the mitochondria contributes to the alteration of various macromolecules including DNA, proteins and lipids as well as impairment of mitochondrial electron transport chain (Cornelius et al., 2013). Excessive generation of ROS derived from mitochondria exacerbates secondary brain injury such as aggravation of neuronal necrosis and apoptosis (Cheng et al., 2012; Cornelius et al., 2013). In addition, a novel mitochondrial ROS-dependent form of programmed cell death, ferroptosis, has been reported recently to be involved in postinjury after TBI (Xie B. S. et al., 2019). Recently, numerous correlative studies have been suggested that Sig-1R directly participated in ameliorating ROS-mitochondrial dysfunction-induced pathological oxidative cell damage through regulating production of mitochondrial ROS and transduction of ROS-induced downstream signaling pathways. Previous study reported that pretreatment of Sig-1R agonist SA4503 in primary cortical neuron cultures effectively protected neurons against H_2_O_2_-induced cell death (Tuerxun et al., 2010). It was shown that the process which LPS-induced retinal microglia release of inflammatory mediators and ROS, is significantly suppressed by Sig-1R agonist (+) pentazocine, however, these inhibitory effects were abolished by Sig-1R antagonist BD1063 (Zhao et al., 2014). Similarly, pentazocine was also demonstrated to protect human lens cell against oxidative stress-induced apoptotic cell death (Wang et al., 2012). In LPS-stimulated BV2 microglia, Sig-1R agonist SKF83959 significantly decreased the release of pro-inflammatory mediators and production of ROS, and these effects were blocked in the presence of Sig-1R antagonist (BD-1047 or BD-1063) (Wu et al., 2015). In the mouse model of AD, it was reported that Sig-1R agonist OZPOO2 significantly attenuated Aβ_25__–__35_-induced hippocampal ROS increase (Maurice et al., 2019). Furthermore, *in vitro* H_2_O_2_-induced ROS concentration model, it was detected that Sig-1R directly interacted with brain Zinc finger protein 179 (Znf179) and regulated its expression, and overexpression of Znf179 exerted similar neuroprotective effect to that of Sig-1R agonists on ROS-induced damage in cells (Su et al., 2016). Paradoxically however, Sig-1R agonist (+) pentazocine was reported to promote NADPH-dependent production of ROS in the brain mitochondria (Natsvlishvili et al., 2015). Furthermore, in rat chronic constriction injury (CCI) model of neuropathic pain, Sig-1R agonist PRE-084 effectively potentiated the activation of Nox2 and production of ROS in spinal cord, whereas these synergistic effects were blocked by antagonists BD-1047 or apocynin (Choi et al., 2013). One may wonder why Sig-1R exerts such dual effects on regulating production of ROS, and a recent study well explains this question. By incubating mouse crude mitochondria preparation, it was reported that Sig-1R agonists promoted production of ROS and enhanced activity of Complex I under physiological condition, whereas they also effectively attenuate Aβ_1__–__42_-induced increase in ROS and counteracts the effects that Aβ_1__–__42_-induced Complex I and IV dysfunctions (Goguadze et al., 2019). This study makes it reasonable that activation of Sig-1R in spinal cord promoted ROS production, as moderated ROS concentration in spinal cord has been reported to be critical for central sensitization against acute and chronic nociception (Meeus et al., 2013). Inasmuch as moderate ROS concentration is critical for regulating cellular responses and functions under physiological conditions, whereas under pathological conditions, excessive production of ROS exhibits deleterious damage to cells, we speculate that Sig-1R also plays Ying-Yang role in regulating production of ROS, and specific mechanism of actions by which Sig-1R acts as a ROS promotor or a scavenger is dependent on given conditions. Overexpression of Sig-1R in COS-7 cells were detected to effectively decrease the production of ROS comparing to mock COS-7 cells (Pal et al., 2012). Further investigation of this study revealed that activation of Sig-1R promotes antioxidant protein peroxiredoxin 6 (Prdx6) expression and activates Antioxidant Response Elements(ARE)to increase mRNA expression of NADPH quinone oxidoreductase 1(NQO1) and superoxide dismutase (SOD) (Pal et al., 2012). In addition, recent study found that in the primary retinal Müller glial cells, in which increased ROS levels were measured in retinal Müller cells that isolated from Sig-1R-KO mouse compared to that from WT mouse accompanied with significant decreases in expression of antioxidant proteins including SOD1, NQO1, catalase (CAT), hemeoxygenase-1 (HMOX1), glutathione-S-transferases (GST) and glutathione peroxidases (GPX) (Wang et al., 2015). In response to the ROS concentration, nuclear factor erythroid 2-related factor 2 (Nrf2) is freed from restriction of Kelch like-ECH-associated protein 1 (KEAP1) and subsequently transfers into nucleus where it directly binds to ARE, triggering a series of transcriptional processes which are responsible for encoding antioxidant proteins (Sies et al., 2017). It was demonstrated that Sig-1R functionally regulated ROS by modulating the pathway of Nrf2-KEAP1 and affinity of Nrf2-ARE binding (Wang et al., 2015). A recent study observed that in cultured astrocytes, addition of Sig-1R agonist SA4503 protected them against LPS-induced production of NO and ROS by upregulating expression of Nrf2 and HO-1 (Wang and Zhao, 2019). Taking these findings together, we conclude that under different pathophysiological conditions, Sig-1R plays a pivotal role in regulation of mitochondrial ROS concentration mainly through Nrf2-KEAP1 pathway. Although it was reported that Sig-1R agonist PRE-084 reduced nitrosative and oxidative stress after TBI (Dong et al., 2016), the specific underlying mechanism by which Sig-1R combats TBI-induced mitochondrial ROS accumulation remain to be totally clarified. These lines of evidence above may provide critical clues to the further investigation on pathophysiological mechanism of action which Sig-1R regulates mitochondrial ROS following TBI.

## Sigma-1 Receptor Suppresses Glutamate Excitotoxicity by Regulation of *N*-Methyl-*D*-Aspartate Receptor Activities

Excitotoxicity as one of the essential hallmark pathological events following secondary brain injury is a primary contributing factor in neuronal death and acute cell swelling after TBI (Park et al., 2008; Chamoun et al., 2010). Accumulation of extracellular glutamate provokes two main glutamatergic receptors on the synaptic terminal: (1) triggering rapid influx of calcium into cytoplasm and (2) activating a series of pro-apoptosis cascades (Park et al., 2008). These two glutamatergic receptors are α-amino-3-hydroxy-5-methyl-4-isoxazole-propionic acid receptors (AMPARs) and *N*-methyl-D-aspartate receptors (NMDARs; Meldrum, 2000). Both receptors are glutamate-gated ion channels, in which AMPARs are primarily involved in regulating Na^+^ influx that depolarizes the postsynaptic neuron, whereas NMDARs are responsible for the calcium influx that modulates cellular signal transduction (Bell et al., 2009). The capacity of Sig-1R to modulate synaptic transmission and plasticity in the brain has been well established (Kourrich et al., 2012). Previous studies have shown that Sig-1R regulated neuronal excitability and synaptic function mainly by affecting NMDAR activities rather than AMPAR activities (Fletcher et al., 1995; Bergeron et al., 1996; Snyder et al., 2016).

**FIGURE 3:**
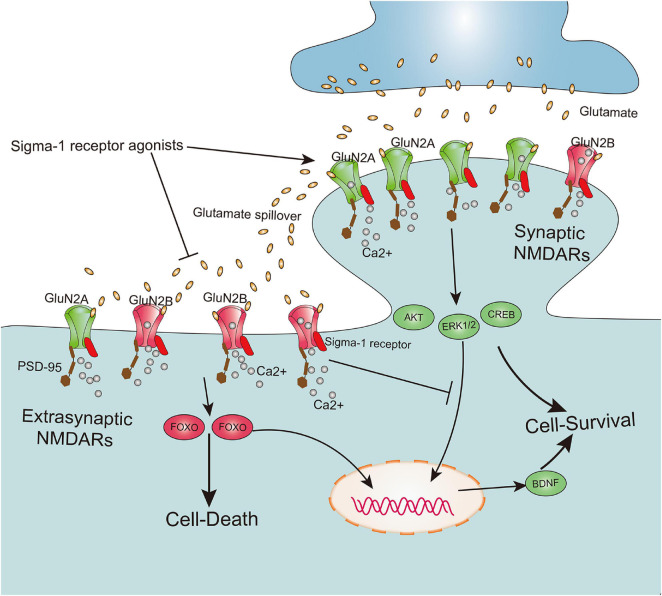
Sigma-1 receptor determines cell fate by modulation extrasynaptic NMDARs and synaptic NMDARs activities. The subunit GluN2A and GluN2B, respectively, are mainly existed on synaptic and extrasynaptic regions. Both synaptic NMDARs and extrasynaptic NMDARs contribute to calcium transients which trigger a series of nuclear calcium-dependent signaling pathways. They lead to totally opposing biochemical processes and cellular consequences, in which activation of synaptic NMDARs promote cell survival by upregulation of the expression of extracellular signal-regulated kinase 1/2 (ERK1/2), protein kinase B (Akt), cAMP response element-binding protein (CREB) and brain-derived neurotrophic factor (BDNF), whereas excessive activation of extrasynaptic NMDARs lead to cell death by contribution to CREB shut-off, inactivation Akt and ERK1/2, and activation of pro-death transcription factor forkhead box protein O (FOXO). Agonism of sigma-1 receptor not only promotes the synaptic NMDARs activities, but also inhibits extrasynaptic NMDARs activities.

According to the localization of NMDARs at the synapse, these receptors are mainly classified as synaptic NMDARs and extrasynaptic NMDARs (Rao and Craig, 1997). Although both are responsible for calcium transients that trigger a variety of nuclear calcium-dependent signaling pathways, they cause totally opposite biochemical processes and cellular consequences. Furthermore, activation of synaptic NMDARs implicated in upregulating the expression of extracellular signal-regulated kinase 1/2 (ERK1/2), protein kinase B (Akt), cAMP response element-binding protein (CREB), and brain-derived neurotrophic factor (BDNF) exert positive physiological effects on intense synaptic plasticity as well as neuronal survival, development, and function. However, excessive activation of extrasynaptic NMDARs contribute to CREB shut-off, inactivation of Akt and ERK1/2, and activation of pro-death transcription factor forkhead box protein O (FOXO), subsequently leading to neuronal excitotoxicity and death (Zhang et al., 2007; Hardingham and Bading, 2010; Lai et al., 2014) (Figure 3). Meanwhile, it has been shown that a cell’s fate may be not solely dependent on the degree of NMDAR activation, but also on subcellular distribution of NMDARs at the synapse and its subunit composition (Hardingham et al., 2002; Parsons and Raymond, 2014). Recently, underlying bidirectional effects of the Sig-1R on NMDARs have been reported, in which activation of the Sig-1R may not only exert positive effects on neuronal synaptic plasticity and neuronal survival by potentiating functions of synaptic NMDARs, but also alleviate neuronal excitotoxicity and death by negatively regulating extrasynaptic NMDARs (Figure 3). One pioneering study on learning and memory disorders reported that the Sig-1R agonist PRE-084 significantly ameliorated learning impairments (Maurice et al., 1994). Further evidence has also confirmed this correlation between the Sig-1R and neuronal synaptic plasticity and survival, elucidating a mechanism by which positive effects of several Sig-1R synthetic ligands such as SKF10047 (Zou et al., 1998), (+)pentazocine (Martina et al., 2007), SA4503 (Maurice and Privat, 1997; Zou et al., 2000), and PRE-084 (Pabba et al., 2014) contribute to enhancement of neuronal synaptic plasticity and ameliorate cognitive deficits.

The studies above have mainly focused on the positive effects of Sig-1R on NMDARs in the hippocampus, whose function is related to memory, learning and cognition. However, activation of Sig-1R has also been reported to exert a negative effect on NMDARs in the central and peripheral nervous systems, preventing glutamate excitotoxicity-induced neuronal death. One study reported that Sig-1R ligands such as JO1784 and (+) pentazocine significantly inhibited NMDAR activities and NMDAR-induced calcium influx in primary cultured rat frontal cortical neurons (Hayashi et al., 1995). Specifically, Sig-1R ligands ifenprodil or haloperidol effectively rescued dopaminergic neurons in midbrain slice cultures that were exposed to excessive NMDA from degeneration and death, and Sig-1R selective agonist SKF10047 also significantly protected dopaminergic neurons against NMDA excitotoxicity (Shimazu et al., 2000). In addition, it was found that Sig-1R agonist SKF10047 directly inhibited NMDAR response and attenuated glutamate neurotoxicity, alleviating chemical ischemia-induced neuronal death *in vitro*, whereas these neuroprotective effects were not abolished by the Sig-1R antagonist (Kume et al., 2002). NMDARs are also abundantly expressed on RGCs (output neurons of the retina), and are largely distributed at extrasynaptic areas of GCs (Zhang and Diamond, 2006). It has been further demonstrated that NMDA or glutamate-induced excessive activation of NMDARs contribute to RGC excitotoxicity and death, and that the administration of Sig-1R agonists significantly suppressed these pathological alterations, implicating possible negative outcomes of Sig-1R activity on extrasynaptic NMDARs (Dun et al., 2007; Zhang et al., 2011; Zhao et al., 2016). These findings therefore indicate dual effects of Sig-1Rs, in which Sig-1Rs may facilitate impairment of synaptic NMDARs, which are related to neuronal synaptic plasticity and survival, whereas in response to pathological nerve injury events, Sig-1Rs possibly inhibit excessive extrasynaptic NMDAR responses that determine neuronal excitotoxicity and death.

It is widely accepted that the bidirectional effects of the Sig-1R on NMDARs may depend on the different pathophysiological conditions and type-specific and function-specific neurons in the CNS and peripheral nervous system (PNS). However, the specific mechanism by which the Sig-1R exerts bidirectional effects on NMDARs still remains to be clarified. It has long been known that most of the NMDARs in the CNS are typically assembled as di-heteromers of GluN1/GluN2A or GluN1/GluN2B, or as a tri-heteromer of GluN1/GluN2A/GluN2B (Paoletti et al., 2013; Parsons and Raymond, 2014). The concept that the subunits GluN2A and GluN2B mainly exist on synaptic and extrasynaptic regions, respectively, remains controversial. Meanwhile, it has been suggested that differences in subunit composition contribute to opposite pathophysiological events, in which activation of GluN2A/synaptic NMDARs promote cell survival, while agonism of GluN2B/extrasynaptic NMDARs lead to cell death (Zhang et al., 2007; Hardingham and Bading, 2010; Karpova et al., 2013; Parsons and Raymond, 2014) (Figure 3). Interestingly, a recent study using an AFM imaging assay in recombinant cells and proximity-ligation assay *in vivo* found a direct interaction between the Sig-1R and NR1 subunit of NMDARs (Balasuriya et al., 2013). Moreover, an *in vivo* study of the rat hippocampus revealed that Sig-1R agonists significantly promoted the expression of both the GluN2A and GluN2B subunit of NMDARs, as well as the postsynaptic density protein 95 (PSD-95; Pabba et al., 2014). This study further demonstrated that the Sig-1R directly binds to GluN2 subunits at the ER and mediates delivery of the NMDAR/PSD-95 complex from the ER to the plasma membrane (PM), and that these events can be well promoted by Sig-1R agonists such as SKF-10047 and PRE-084 (Pabba et al., 2014). We therefore speculate that the Sig-1R directly regulates the expression of NMDARs and PSD-95, as well as performs as a chaperoning scaffold, mediating the NMDAR shift from the ER compartment to the PM surface. However, in study with using Co-IP experiments, it was also found that Sig-1R agonists effectively disrupted the interaction between GluN2B and PSD-95 (Pabba et al., 2014). Since PSD-95 directly binds to both GluN2A and GluN2B and mediates downstream signaling, decreasing interaction between GluN2 and PSD-95 has been reported to attenuate excitotoxic damage and cell death, without influencing synaptic NMDA currents (Aarts et al., 2002). Taking these findings together, we hypothesize that the Sig-1R may exert its bidirectional effects by means of regulation of NMDAR subunit expression and coordination of the shift between synaptic NMDARs and extrasynaptic NMDARs on the postsynaptic surface. It may also further influence the interaction between NMDARs and membrane-associated guanylate kinases such as PSD-95 (Figure 3). Quantificational and permanent glutamate-induced synaptic NMDAR activities are essential for the CNS to maintain neuronal communication and long-term potentiation (LTP) under physiological conditions, whereas following pathological nerve injury such as TBI, the massive yet transient glutamate spillover that contributes to overactivation of NMDARs leads to extrasynaptic NMDAR-induced neuronal excitotoxity and death (Parsons and Raymond, 2014). Interestingly, many clinical trials and preclinical animal experiments have demonstrated that pathologically accumulative glutamate outside the synapsis triggers hyperactivation of the NMDARs (possibly extrasynaptic NMDARs) only during the acute phase following TBI, leading to a series of deleterious downstream signaling cascades and neuronal death. Subsequently, functions of NMDARs mainly related to synaptic NMDARs are gradually and persistently attenuated in the subacute period of postinjury, further causing detrimental outcomes such as cognitive deficits (Biegon et al., 2004, 2018; Shohami and Biegon, 2014).

As activation of the Sig-1R has been reported to create positive effects on synaptic NMDARs and negative effects on extrasynaptic NMDARs, it may provide another insight into the Sig-1R therapeutic effect on glutamate excitotoxicity-induced neuronal death during the acute and/or subacute period following TBI. This is especially true since studies investigating the Sig-1R in NMDARs following TBI are extremely limited, and the specific mechanism by which the Sig-1R regulates synaptic/extrasynaptic NMDARs in different periods following TBI is also unknown.

## Sigma-1 Receptor Modulates the Intracellular Calcium Homeostasis

Intracellular calcium homeostasis plays broad spectrum of roles in modulating and maintaining functions such as neuronal excitability, cell migration, cell metabolism, gene transcription and cell survival/death (Berridge et al., 2000; Giorgi et al., 2018). After TBI, excessive extracellular calcium influx and intracellular ER calcium efflux contribute to ER stress, mitochondrial dysfunction and cytosolic calcium overloaded, leading to concomitant neuronal excitotoxicity and death (Weber, 2012; Ruiz et al., 2018). A large number of findings has been evidenced that Sig-1R played critical role in determining calcium concentration and cell fate. In addition to modulation of NMDARs activity, Sig-1R has also been reported to affect intracellular calcium concentration through modulation of other calcium transport channels or receptors (Figure 2).

### Sigma-1 Receptor Regulates the Voltage-Gated Calcium Channels Activities

Voltage-gated calcium channels (VGCCs) have been demonstrated to be broadly expressed in various excitable cells such as neurons (Timmermann et al., 2002). In response to membrane depolarization, VGCCs serve as transporters of extracellular calcium influx, transducers of downstream calcium signaling, and molecular switches of presynaptic neurotransmitter release (Atlas, 2013; Zamponi, 2016). Multifarious types of VGCCs play diverse and characteristic roles in neurons according to their uniquely subcellular localization, for example, most of L-type voltage-gated calcium channels (L-VGCCs) are positioned at neuronal cell bodies and dendrites, and predominantly regulate neuronal plasticity and extracellular calcium entry, whereas N-type, R-type and P/Q-type VDCCs are mainly involved in regulating neurotransmitter release from presynaptic terminals (Yagami et al., 2012). Recently, numerous studies have proposed that inhibition of both L-VGCCs and N-VGCCs sufficiently rescued excessive calcium influx-induced neuronal apoptosis and cognitive dysfunction following TBI (Ercan et al., 2001; Lee et al., 2004; Gibson et al., 2010; Shahlaie et al., 2010; Gurkoff et al., 2013). It has been reported in previous findings that Sig-1R was implicated in regulating the functions of multiple subtypes of VGCCs (Church and Fletcher, 1995; Zhang and Cuevas, 2002). Sig-1R agonists such as (+) pentazocine and DTG [1,3-di-(2-tolyl) guanidine] could significantly dampen calcium current of sensory neurons by directly inhibiting activation of VGCCs, whereas the effect of Sig-1R agonists on inhibition of VGCCs was abolished by Sig-1R antagonist BD1063 (Pan et al., 2014). It was reported that in purified RGCs, stimulation of Sig-1R by its both agonists [SKF10047 and (+)-pentazocine] significantly attenuated calcium influx which was mediated by KCl-induced activation of L-VGCCs, whereas the effect of Sig-1R agonists on inhibition of calcium influx was blocked in the presence of Sig-1R antagonist (BD-1047) (Mueller et al., 2013). With using immunoprecipitation technique, a further finding discovered that there was a direct protein-protein interaction between Sig-1R and L-VGCCs, and Sig-1R agonist SKF10047 could inhibit calcium currents in rat primary retinal ganglion cell line-5(RCGs-5) (Tchedre et al., 2008). However, it is contradictory that pregnenolone sulfate (PREGS) as an agonist of Sig-1R facilitated activation of L-VGCCs and subsequent increase of calcium currents in the CA1 neuronal synapses of hippocampus, promoting the induction of presynaptic plasticity and L-VGCCs-dependent LTP (long-term potentiation) (Sabeti et al., 2007). Since L-VGCCs exert specific function in unique region of neuron, we hypothesize that Sig-1R ligands may also exert dual effects on L-VGCCs-mediated calcium influx depending on neuronal regions. Beyond the suppressive effect on L-VGCCs, Sig-1R has also been demonstrated to be involved in regulating N-VGCCs. Previous study observed that Sig-1R agonist igmesine was implicated in modulating extracellular calcium influx by regulating the activities of both L-VGCCs and N-VGCCs (Urani et al., 2002). It was also reported that activation of Sig-1R by SKF10047 attenuated 4-AP-induced glutamate release in rat cerebral cortex nerve terminals, and the inhibitory mechanism was associated with decrease of calcium influx through N-VGCCs and P/Q-VGCCs (Lu et al., 2012). In addition, application of Sig-1R agonist SA4503 in primary cultures of hippocampal neurons effectively promoted axon outgrowth which was mainly associated with both L-VGCCs and N-VGCCs, and further investigation of this study revealed that Sig-1R agonist SA4503 directly regulated activities of both channels (Li et al., 2017). Recently, using fluorescence resonance energy transfer (FRET) and co-immunoprecipitation (Co-IP) assays, an in-depth experiment identified the mechanism, by which Sig-1R inhibitory action on N-VGCCs was established on the basis of constitutively physical interaction between them; Sig-1R agonist promoted the conformational alteration in the chaperone - channel complex that depresses calcium influx through N-VGCCs (Zhang et al., 2017). These findings above may provide a novel insight into Sig-1R therapeutic effect on TBI, revealing that principally negative modulation on both L-VGCCs and N-VGCCs by Sig-1R ligands may protect neurons against immoderate calcium influx-induced apoptosis and provide a neuroprotective event following TBI.

### Sigma-1 Receptor Regulates Non-voltage-Gated Calcium Channels Activities

Releasing calcium from ER to mitochondrial is an important way to regulate intracellular calcium homeostasis and mitochondrial metabolism, as well as ATP production and apoptosis process (Giorgi et al., 2018). The primary channel that participates in regulation of calcium transport between ER and mitochondria at MAMs is IP3R3-GRP75-VDAC1 complex (Bartok et al., 2019; Figure 2). Upon the ER stress, prolonged ER calcium depletion or ligand stimulation, Sig-1R dissociates from BiP and subsequently binds to IP3Rs and stabilizes them, effectively facilitating calcium transfer from ER into mitochondrial (Cardenas et al., 2010; Figure 2). It was shown that deficiency of Sig-1R significantly led to abnormal intracellular calcium concentration through IP3R when stimulated with bradykinin, whereas calcium was depleted in the ER after stimulating cell with ionomycin, indicating abnormal modulation of calcium transport in the absence of Sig-1R (Prause et al., 2013).

In response to depletion of ER calcium store, ER membrane calcium sensor stromal interaction molecules 1 (STIM1) initiates oligomerization and then redistributes to the ER-PM contact site, where it associates with Orai1 and provokes its channel activity, ultimately activating mechanism of store-operated calcium entry (SOCE) and restoring ER calcium homeostasis (Prakriya and Lewis, 2015). It has been reported that Sig-1R is involved in regulating the calcium influx through SOCE pathway (Figure 2). Cocaine as an agonist of Sig-1R, sufficiently inhibited SOCE in rat brain microvascular endothelial cells, whereas its inhibitory effect was counteracted by selective Sig-1R antagonist BD-1063 or NE-100 (Brailoiu et al., 2016). It was discovered in further study that Sig-1R could interact with STIM1 and inhibit it coupling to Orai1, attenuating SOCE-mediated calcium influx. The Sig-1R agonist, SKF10047, effectively lessened interplay between Sig-1R and STIM1 and reduced intracellular calcium influx, whereas deficiency of Sig-1R or using antagonist performed reversed effects (Srivats et al., 2016).

Moreover, several findings have also shown that stimulation of Sig-1R depressed activity of acid-sensing ion channel a (ASIC1a), which successively activated downstream intracellular calcium influx signaling (Herrera et al., 2008; Carnally et al., 2010).

Consequently, there may be a sophisticated and coordinated mechanism by which Sig-1R exerts a direct or indirect control, facilitatory or inhibitory effects on maintenance of intracellular calcium homeostasis (Figure 2). The specific mechanisms of correlation between Sig-1R and intracellular calcium homeostasis is gradually discovered by increasing studies, and clarification of these mechanisms may provide a promising therapeutic target for treatment of intracellular calcium mobilization-induced secondary brain injury after traumatic brain injury.

## Sigma-1 Receptor Plays an Important Role in Regulation of Neuroinflammation

Neuroinflammation primarily orchestrated by activated microglia and astrocytes is well-established as a hallmark of secondary brain insult after TBI and has been considered a primary contributing factor in chronic brain damage following TBI (Johnson et al., 2013; Karve et al., 2016). It has been demonstrated in previous studies that the Sig-1R is broadly expressed in microglia and astrocytes (Ruscher et al., 2011; Francardo et al., 2014), and regulates these two glia-mediated neuroinflammation in a variety of CNS disease (Griesmaier et al., 2012; Francardo et al., 2014; Peviani et al., 2014).

### Sigma-1 Receptor Regulates Astrocyte-Mediated Neuroinflammation

It has been found that pretreatment of the Sig-1R antagonist BD-1047 suppresses methamphetamine-induced activation of primary rat astrocytes and upregulation of Sig-1R expression (Zhang et al., 2015). In addition, methamphetamine fails to increase the GFAP expression in the primary astrocyte cultures derived from Sig-1R KO mice (Zhang et al., 2015). In a mouse model of motor neuron degeneration, chronic administration of PRE-084 inhibited the pathological astrocytosis (Peviani et al., 2014). A different study found that astrocytosis was enhanced in neuron-astrocyte mixed cultures derived from Sig-1R KO mice compared with WT mice (Weng et al., 2017), and iNOS and TNFα expression levels were reduced by SA4503 treatment (Wang and Zhao, 2019) in LPS-treated astrocytes.

### Sigma-1 Receptor Regulates Microglia-Mediated Neuroinflammation

Microglia as the primary resident immune sentinels in the CNS exert dual effects depending on their M1/M2 phenotype during neuroinflammation (Gordon and Taylor, 2005; Hu et al., 2015; Xiong et al., 2016; Li X. et al., 2020). Promoting microglia transformations from the M2 to M1 phenotype is a pivotal therapeutic target for amelioration of neuroinflammation following TBI. In newborn mice, PRE-084 was found to suppress microglia activation at the lesion site (Griesmaier et al., 2012), and chronic PRE-084 treatment reduced microglial immunoreactivity in the ventral horn of SOD1 mice (Mancuso et al., 2012) in an ALS model. Similarly, in a mouse model of Parkinsonism, it was demonstrated that chronic PRE-084 treatment effectively attenuated 6-hydroxydopamine lesion-induced microglia activation in the striatum and substantia nigra (Francardo et al., 2014). An *in vitro* study reported that Sig-1R activation inhibited the migration and inflammatory responses of microglia activated by LPS (Hall et al., 2009), and Sig-1R agonist SKF83959 has also been found to suppress the release of pro-inflammatory cytokine, such as TNF-α, IL-1β, and iNOS from microglia activated by LPS (Wu et al., 2015). A different study demonstrated that (+) pentazocine inhibited LPS-evoked retinal microglia activation primarily through the MAPK/ERK pathway (Zhao et al., 2014), and a mouse model of spinal muscular atrophy found that PRE-084 administration suppressed reactive gliosis and corrected the M1/M2 phenotype imbalance (Cervero et al., 2018). Furthermore, administration of PRE-084 after embolic stroke significantly suppresses release of pro-inflammatory factors and promoted secretion of anti-inflammatory cytokines, such as IL-10 and IL-4 (Allahtavakoli and Jarrott, 2011), and a recent mouse model of TBI showed that PRE-084 successfully suppressed microglia activation, attenuated brain edema, and improved neurological function after TBI (Dong et al., 2016).

Collectively, these findings strongly indicate that the Sig-1R may play a key role in the regulation of neuroinflammation in various neurological disorders including TBI.

## Sigma-1 Receptor Plays an Important Role in Neurodegenerative Diseases

Neurodegenerative diseases are described as disorders with the gradual loss of the structure or function of the neurons, including Alzheimer’s disease, Parkinson’s disease, Huntington disease etc. (Soto and Pritzkow, 2018; Shetty et al., 2019; Xie F. et al., 2019; Zhou H. et al., 2020). Patients after brain trauma have long-term neurodegenerative related symptoms, which seriously threatens the patient’s quality of life. Dysfunction of mitochondria-associated-ER membrane and accumulation of abnormal protein folding are major pathological mechanism common to neurodegenerative diseases (Herrando-Grabulosa et al., 2021). There are a lot of evidence that Sig-1R plays a key role in multiple neurodegenerative diseases.

### Sigma-1 Receptor in Alzheimer’s Disease

The main pathological features of AD include deposition of neurofibrillary tangles of the abnormal tau protein and Plaque deposition composed of β-amyloid (Aβ) peptides (Li W. et al., 2020; Wong et al., 2020). Emerging evidence has been demonstrated that Aβ is produced intracellular at MAMs and affects the structure and function of ER, mitochondria and MAMs (Schreiner et al., 2015). Sig-1R agonist Afobazole could significantly prevented the increase of calcium induced by Aβ (Behensky et al., 2013). The loss of Sig-1R was found in brains and postmortem tissues of AD patients (Hedskog et al., 2013). As mentioned above, Sig-1R agonism effectively protected neurons against Aβ toxicity and ameliorate the learning and memory deficits in AD mice. Knockout of Sig-1R significantly upregulate the oxidative stress andexacerbates memory deficits in a mice model of AD (Lahmy et al., 2014). In addition, certain genetic combination of Sig-1R and apolipoprotein E (APOE) genotypes synergistically increase the risk of AD (Huang et al., 2011). Recent studies found that Sig-1R functionally interacts with presenilin 1 (PS1) and presenilin 2 (PS2) at MAMs, which are implicated in AD (Ryskamp D. A. et al., 2019). It was reported that Sig-1R agonist Predopidine maintains the ER calcium homeostasis by reducing luminal calcium in cultured hippocampal neurons conditional PS double-knockout mice (Ryskamp D. et al., 2019). Loss of mushroom spines may be the anther underlying pathogenesis of AD. Sig-1R agonists can promote neurogenesis in the hippocampus (Ryskamp D. et al., 2019) and may reduce memory impairment because they can stabilize mushroom spines, which are the location of robust synaptic connections that encode lasting information (Hayashi-Takagi et al., 2015). In addition, Sig-1R agonists were found to reduce mushroom spines loss in hippocampal cultures prepared from presenilin-1-M146V knock-in (PS1-KI) mice (Ryskamp D. et al., 2019).

### Sigma-1 Receptor in Parkinson’s Disease

Parkinson’s disease (PD) is a progressing neurodegenerative disorder characterized by motor dysfunction caused by selective degeneration of dopaminergic neurons in the substantia nigra pars compacta and abnormal accumulation of α-synuclein protein called Lewy bodies (Xie F. et al., 2019; Gao et al., 2020; Wang T. et al., 2020; Yang et al., 2020; Zhou H. et al., 2020). Sig-1R is expressed in dopaminergic neurons and modulates dopamine transporter conformation and cocaine binding (Hong et al., 2017). The downregulation of Sig-1R was also detected by PET studies in putamen of PD patients (Toyohara et al., 2009). Similar to PD patients, Sig-1R KO mice also showed age-related deficits in motor behavior and dopaminergic neurons death and degeneration (Hong et al., 2017). Recent studies have demonstrated that pharmacological stimulation of the Sig-1R protect shows neuroprotective in PD models. In a mouse model of PD, it was found that Sig-1R agonist PRE-084 for 5 consecutive weeks significantly restore the mice behaviors deficits (Francardo et al., 2014). In a unilateral 6-hydroxydopamine (6-OHDA) lesion model of parkinsonism in mice, it was found that daily administration of a low dose pridopidine significantly promotes mice behavior recovery and upregulates the expression of BDNF, GDNF, and the phosphorylated ERK1/2 (Francardo et al., 2019). In a 6-OHDA model of Parkinson’s disease, it was demonstrated that administration of Sig-1R agonists afobazole or PRE-084 over 2 weeks significantly restores the motor deficits and inhibit decreases of dopamine in the 6-OHDA-lesioned striatum, whereas these protective effects were blocked by antagonists BD-1047 (Voronin et al., 2019; Kadnikov et al., 2020). In a Dicer conditional knockout mouse model of Parkinson’s disease, PRE-084 were found to attenuate the dopaminergic neurons loss and behavioral abnormalities (Guo et al., 2020). In a further investigation of pathological development of 1-methyl-4-phenyl-1,2,3,6-tetrahydropyridine (MPTP)-induced mouse model of PD, administration of PRE-084 significantly rescued dopaminergic neurons loss by regulating mitophay (Wang M. et al., 2020). A recent study revealed that concomitant administration of a7 subtype of nicotinic acetylcholine receptor (a7-nAChR) agonist and Sig-1R agonist exert neuroprotective and anti-inflammatory effects in a 6-OHDA model of PD (Vetel et al., 2021).

### Sigma-1 Receptor in Huntington Disease

Huntington disease (HD) is a devastating autosomal dominant caused by a repeat expansion in polyQ mutant huntingtin protein (mHtt), resulting in progressively disabling motor and cognitive deficits. Recent clinical trials have reported that Sig-1R agonist Pridopidine has neuroprotective efficacy in treating motor deficits of HD patients (Lundin et al., 2010; Huntington Study Group, 2013; Reilmann et al., 2019). A recent PET study provided significant clarification that Pridopidine acts as a selective Sig-1R agonist showing almost total Sig-1R occupancy with negligible occupancy of the dopamine D2/D3 receptor in HD patients (Grachev et al., 2021). Upregulation of Sig-1R accompanied by dysregulation of ER calcium was detected both HD mice and HD patients (Ryskamp D. et al., 2017). Large-scale transcriptomic analysis of difference between YAC128 HD mice and wild-type mice revealed that Pridopidine also has broad neuroprotective efficacy in restoring transcriptomic disturbances in the striatum, particularly involing synaptic transmission and triggering neuroprotective pathways that are damaged in HD (Kusko et al., 2018). In a vitro model of HD, it was found that PRE-084 promotes cell survival and protects the neuronal PC6.3 cells from deleterious effects induced by expression of N-terminal mutant huntingtin proteins (Hyrskyluoto et al., 2013). The Sig-1R as one of the ER chaperones, was found to be involved in degradation of intranuclear inclusions *via* ER-related degradation machinery in a vitro model of HD. In HD R6/2 mouse model, it was demonstrated that Pridopidine improves motor function in pre-symptomatic and symptomatic HD mice (Squitieri et al., 2015). In addition, Pridopidine significantly reduced the size of mHtt aggregates in the striatal tissues of R6/2 mice and exerted neuroprotective and anti-apoptotic roles in R6/2 mice *via* regulation of neuroprotective molecules expression (Squitieri et al., 2015). In a YAC128 cortico-striatal co-culture model of HD, Sig-1R agonists Pridopidine and 3-PPP were reported to prevent calcium dysregulation and synaptic loss (Ryskamp D. et al., 2017). The role of Pridopidine in striatal neurons is mediated by Sig-1R, including stabilizing ER calcium levels, reducing synaptic store-operated calcium entry and upregulating expression of the calcium regulating proteins calbindin 1 and homer 1a. Furthermore, a recent study revealed that Pridopidine rescues mitochondrial function by normalizing mitochondrial complex activity and provides neuroprotective effects in YAC128 HD mice (Naia et al., 2021).

All these findings above generalized from the current research of neurodegenerative diseases reveal that Sig-1R may play a key role in the protection of loss of the structure or function of the neurons. The functions and mechanisms of Sig-1R in neurodegeneration may provide insight into Sig-1R therapeutic efficacy in TBI.

## Conclusion and Future Perspective

**FIGURE 4:**
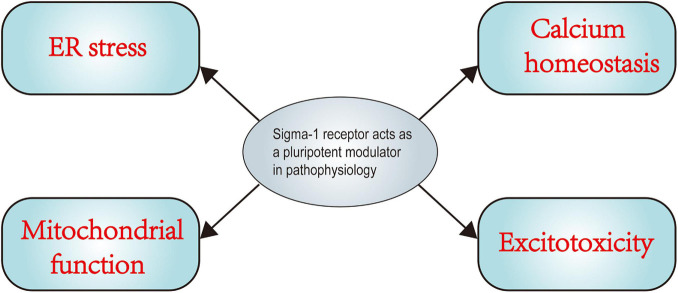
Sigma-1 receptor acts as a pluripotent modulator in pathophysiology. The figure indicates a summary of the mechanism in main pathophysiological processes in which sigma-1 receptor (Sig-1R) plays a key role. Numerous pharmacological studies have confirmed the positive effects of Sig-1R activation on cellular calcium homeostasis, excitotoxicity, ER stress, mitochondrial function. More experiments and further investigations are urgently needed to advance our understanding of Sig-1R and its specific role in traumatic brain injury.

It has been established over the past decades that the Sig-1R acts as a ligand-operated chaperone protein that is primarily located at the MAM. It is precisely because the Sig-1R has versatile ligand affinity, a unique cellular location, and multi-site translocation profile that it acts as a pluripotent modulator in diverse pathological conditions. Recently, emerging evidence suggests that the Sig-1R is involved in pathophysiology of various CNS diseases and exerts multiple neuroprotective effects against nerve damage in various disorders, including TBI. Secondary brain injury-involved ER and oxidative stress, among others, has emerged as a key point in the treatment of TBI. However, a detailed molecular mechanistic account of the Sig-1R in the context of secondary brain injury and pharmacological effects of Sig-1R ligands on nerve injury events and neuroinflammation following TBI is still elusive. Based on a large number of preclinical studies on the neuroprotective effects of Sig-1R in pathological alterations (Figure 4), we speculate that the Sig-1R may be a potential therapeutic target for TBI and pharmacological interventions of the Sig-1R may expand the current narrow therapeutic window of TBI treatment. As research on the roles of Sig-1Rs in TBI is still emerging, many questions remain to be answered before clinical applications can formally begin. For example, why Sig-1Rs can interact with so many structurally diverse proteins and the specific mechanism of the interaction are still unclear. Moreover, it remains to be seen whether Sig-1Rs can interact with other proteins or even organelles to regulate their function. Moreover, while it is known that the Sig-1R can translocate from the MAM to other organelles in response to agonists or stress, it is not known how the Sig-1R acts as a located protein at the MAM to achieve intracellular translocation. Finally, although multiple pathophysiological functions of the Sig-1R have been well established, the specific signal transduction pathways and how they are affected by Sig-1R ligands are still unclear. Further specifically, preclinical studies and clinical trials on the pharmacological effects of Sig-1Rs are therefore necessary in order to expand our current limited therapeutic view of TBI treatment.

## Author Contributions

MS, FC, ZC, WY, and SY searched the biblography and drafted the manuscript. MS prepared the figures. SY, JZ, and XC critically revised the manuscript. All authors contributed to the article and approved the submitted version.

## Conflict of Interest

The authors declare that the research was conducted in the absence of any commercial or financial relationships that could be construed as a potential conflict of interest.

## Publisher’s Note

All claims expressed in this article are solely those of the authors and do not necessarily represent those of their affiliated organizations, or those of the publisher, the editors and the reviewers. Any product that may be evaluated in this article, or claim that may be made by its manufacturer, is not guaranteed or endorsed by the publisher.
